# Case Report: Fascia lata transposition for chronic recurrent perineal hernia followed by laparoscopic colopexy for subsequent rectal prolapse in a dog

**DOI:** 10.3389/fvets.2026.1858804

**Published:** 2026-07-24

**Authors:** Jiyoung Park, Won-Jong Lee, Dae-Hyun Kim

**Affiliations:** 1 Ulsan S Animal Medical Center, Ulsan, Republic of Korea; 2 Department of Veterinary Surgery, College of Veterinary Medicine, Chungnam National University, Daejeon, Republic of Korea

**Keywords:** colopexy, dog, fascia lata transposition, laparoscopy, perineal herniorrhaphy, rectal prolapse, recurrent perineal hernia

## Abstract

This case report describes a surgical management of chronic, recurrent, complicated perineal hernia following bilateral herniorrhaphy in a 6-kg castrated male mixed-breed dog, estimated to be over 10 years old. The patient presented with recurrent right-sided perineal hernia and constipation associated with rectal sacculation. During the herniorrhaphy attempted, severe atrophy of pelvic musculature was identified; therefore, a fascia lata transposition was performed. Postoperatively, the dog developed a rectal prolapse, which was successfully addressed through laparoscopic colopexy. The dog recovered uneventfully, maintaining a good appetite and activity levels. There were no complications related to urination, defecation, gait, or wound healing following either procedure. At the 27-month follow-up, the dog remained in good condition without clinical signs. Fascia lata transposition is effective for complicated perineal hernias lacking of musculature for reconstruction. Laparoscopic colopexy is also a clinically practical option for rectal prolapse in small-breed dogs.

## 1 Introduction

Perineal hernia (PH) occurs when the pelvic diaphragm fails to support the rectal wall due to muscular weakness, leading to the dilatation or deviation of the rectum and the herniation of pelvic or abdominal organs (1, 2). While the exact pathogenesis of PH is not fully understood, it is believed to be multifactorial. Contributing factors include tenesmus associated with constipation, chronic prostatic disease, or rectal disease; myopathy; gonadal hormonal imbalances; the effects of relaxin; and neurogenic atrophy of the pelvic diaphragm muscles (1–3). PH frequently occurs in middle-aged, intact male dogs, with the levator ani muscle being the most commonly affected (2, 4). The severity of PH varies; complicated cases are often associated with recurrence, significant rectal dilatation, concurrent prostatic disease requiring surgery, and retroflexed bladder (5).

Canine PH is primarily a surgical condition requiring reconstruction of the pelvic diaphragm. Various methods are employed, including appositional herniorrhaphy, autografts (such as internal obturator, superficial gluteal, semitendinosus, fascia lata, and tunica vaginalis communis muscles), allografts (canine small intestinal submucosa), xenografts (porcine small intestinal submucosa, porcine dermal collagen), use of synthetic polypropylene mesh or collagen based sponge, and sacroischial sling technique (1, 2, 5–7). In cases of severe atrophy of the levator ani or coccygeus muscles, appositional herniorrhaphy is contraindicated due to excessive tension on the external anal sphincter. Internal obturator muscle transposition (IOMT) is the most widely used technique in such cases, sometimes incorporating the sacrotuberous ligament for lateral repair (3, 5). Organopexy (colopexy, cystopexy, or vasopexy) combined with castration can serve as an effective alternative or adjunctive treatment for PH, yielding satisfactory outcomes by reducing recurrence rates (2, 5, 8).

Rectal prolapse (RP) occurs immediately after herniorrhaphy in 7–42% of cases and is associated with straining due to misplaced sutures in the rectal lumen, postoperative pain, underlying rectal disease, or injury to the external anal sphincter nerve (5, 9). Furthermore, RP may also develop particularly in dogs presenting with bilateral hernias complicated by concurrent rectal sacculation (10). Colopexy is generally performed for chronic RP, and PH to prevent caudal displacement of the colon and rectum (11, 12). This procedure creates a permanent adhesion between the descending colon and the left abdominal wall, reducing the contents within the hernia and preventing recurrent RP (11). While a ventral midline or left paramedian celiotomy is standard, laparoscopic approaches have also been utilized (12). Successful outcomes depend on avoiding suture penetration into the colonic lumen and preventing excessive colonic tension (5, 12). These precautions help prevent complications such as local wound infection and peritonitis, which might necessitate revision surgery (5, 12). Additionally, any underlying causes of straining must be addressed (12).

Fascia lata has been extensively utilized in both human and veterinary medicine for various reconstructive purposes due to its durability, minimal retraction, availability, and low vascularity requirements (13). Autologous fascia lata grafts are frequently used for tendon, ligament, urethral, and joint capsule repairs, as well as abdominal wall reconstructions and cranioplasties (13–15). This report details a fascia lata transposition (FLT) for a complicated PH with rectal sacculation, followed by laparoscopic colopexy for subsequent RP, including the clinical course and long-term prognosis.

## 2 Case description

**Figure 1:**
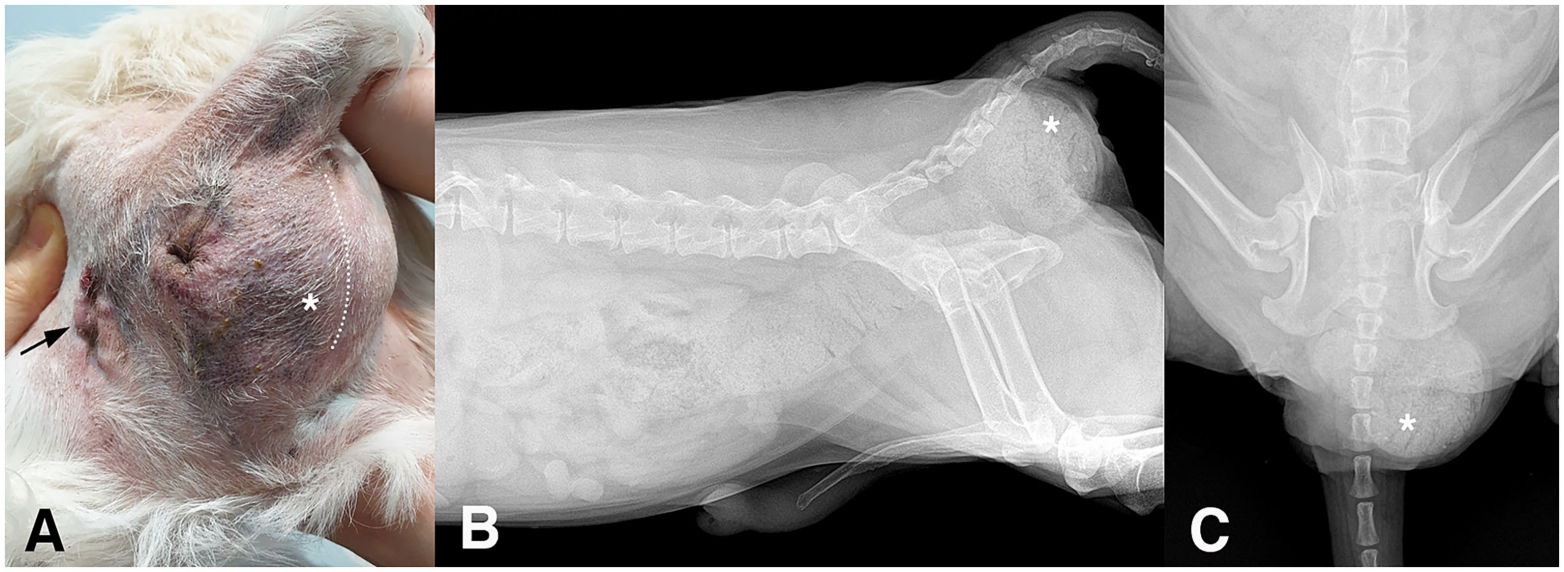
A right perineal bulging **(A)** and fecal impaction in rectal sacculation (**B**: lateral view, **C**: dorsoventral view) of a recurrent perineal hernia in a dog. Black arrow; subcutaneous foreign material, *; herniated rectum, dotted line; previous surgical scar on the right perineum.

A 6-kg castrated male mixed-breed dog, estimated to be over 10 years old, presented for surgical treatment of PH. The dog, rescued 4 months prior, had a body condition score of 6 out of 9 and was bright and alert. Shelter staff reported constipation and a firm, non-painful bulge on the right perineum (Figure 1A). Surgical scars were evident on both sides of the perineum, with subcutaneous foreign material palpable on the left and suture materials on the right. Comprehensive screening tests, including physical examination, rectal palpation, thoracic and abdominal radiography, abdominal ultrasonography, complete blood count, serum chemistry, and electrolytes, revealed no specific findings except for intestinal fecal accumulation and PH with fecal impaction in the rectal sacculation without rectal wall defect or thinning (Figures 1B,C). The patient was initially managed with manual expression and oral lactulose (0.5 mL/kg body weight [BW], twice daily [BID], Dulackhan Easy Syrup, JW Pharmaceutical, Korea) to alleviate tenesmus.

Three weeks later, perineal exploration and herniorrhaphy—initially planned as an IOMT—were attempted under general anesthesia with isoflurane inhalation (< 2%, Ifran, Hana Pharm, Korea) without mechanical ventilation. Premedication and induction were achieved using midazolam (0.2 mg/kg BW, intravenously [IV]; Bukwang Midazolam, Bukwang Pharm, Korea) and propofol (4 mg/kg BW, slow IV; Provive, Pharmbio Korea, Korea), respectively. Cefazolin (20 mg/kg BW, IV; Cefazoline, Chongkundang, Korea) and meloxicam (0.2 mg/kg BW, IV; Metacam, Boehringer Ingelheim Vetmedica Korea, Korea) were administered preoperatively, and a transdermal fentanyl patch (25 μg/h, Durogesic D-trans Patch, Janssen Korea, Korea) was attached 6 h before surgery. Intraoperative fluid therapy was administered using Lactated Ringer’s solution (5 mL/kg/h), and continuous patient monitoring throughout the procedure included heart rate, respiratory rate, pulse oximetry, non-invasive blood pressure, tidal volume, end-tidal carbon dioxide, inspiratory pressure, and body temperature. The isoflurane concentration was adjusted according to the anesthetic depth. The patient was positioned in sternal recumbency with the hind limbs extending beyond the edge of the surgical table. In anticipation of a potential autograft using FLT, the surgical field was extended to include the right caudolateral thigh, which was also prepared aseptically.

**Figure 2:**
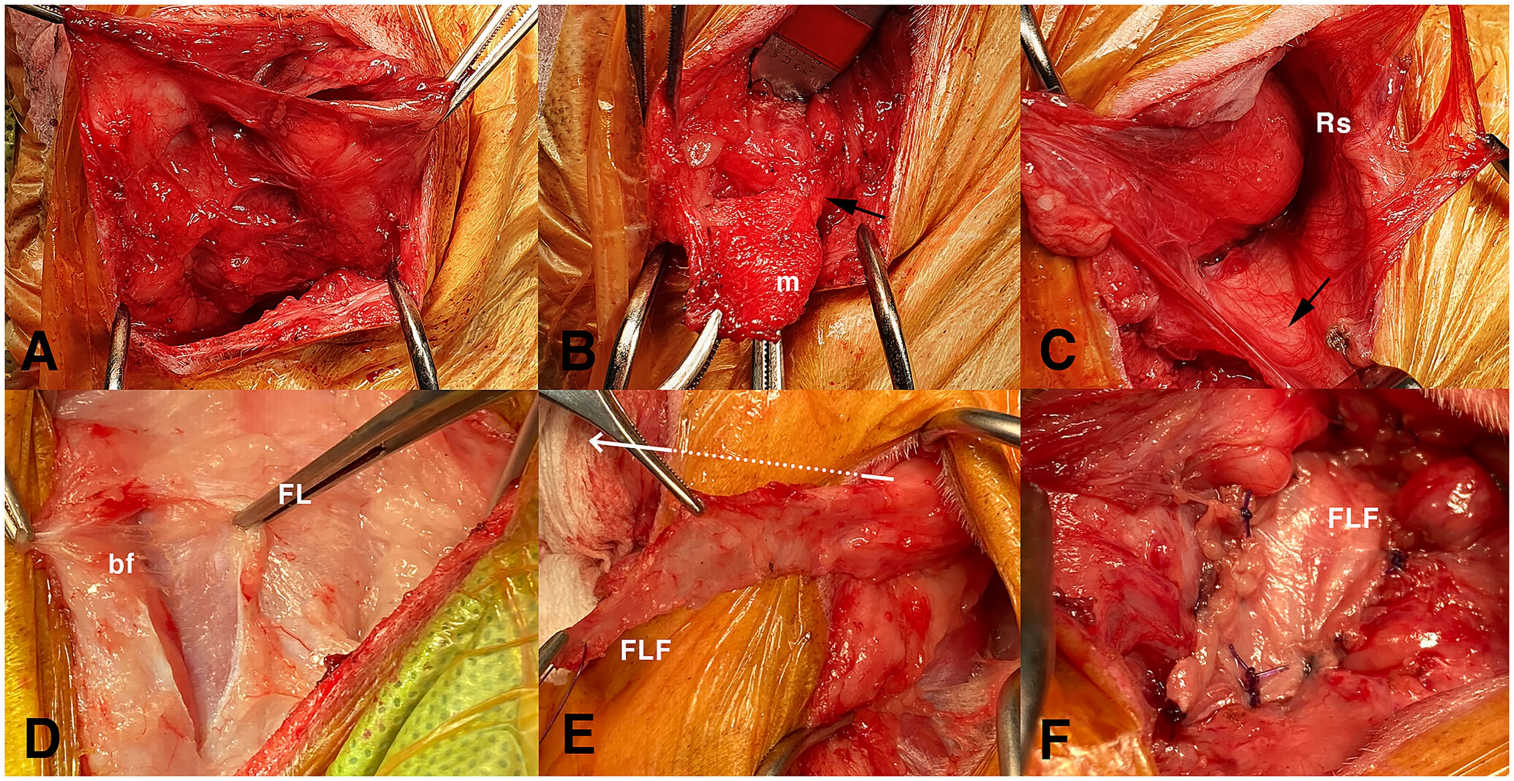
Fascia lata transposition to repair recurrent perineal hernia in a dog. **(A)** Right perineal region showing inflamed tissues with clear, viscous, and slippery discharge, **(B)** previously implanted synthetic mesh (m), **(C)** herniated rectum with sacculation (Rs), **(D)** fascia lata (FL) being dissected, **(E)** fascia lata flap (FLF) before being passed through the subcutaneous tunnel (white arrow), **(F)** completed fascia lata transposition. Black arrows; ischial table, bf; biceps femoris.

Upon exploration of the right perineal region following a skin incision, inflamed subcutaneous tissues, perianal fat, and the hernia sac were found to be interwoven, accompanied by a clear, mildly viscous, and slippery discharge (Figure 2A). During dissection and debridement, a synthetic mesh and non-absorbable suture materials were identified around the ischiatic border and penile root, which were removed along with the inflammatory tissues (Figure 2B). A protrusion of the rectal sacculation, which was the only herniated organ, was also observed without any wall tearing (Figure 2C). The pelvic musculature was challenging to distinguish, with no evident muscular structures identified except for the external anal sphincter. The herniation was bordered by the external sphincter, sacrotuberous ligament, ischiatic table, and sacrum. The internal obturator muscle was barely visible and thus not a viable option for reconstructing the pelvic diaphragm.

An additional incision was made on the right lateral thigh for FLT (Figure 2D). After dissecting the subcutaneous tissue, a fascia lata flap was created. The borders of the flap were defined by the sartorius muscle (cranially), the cranial border of the biceps femoris muscle (caudally), and the patella (distally). Vertical incisions along the cranial and caudal margins were extended proximally to the level of the tensor fascia lata, with the flap remaining attached at its proximal origin. The flap, including the tensor fascia lata, was then rotated caudo-medially and passed through a subcutaneous tunnel into the perineal region (Figure 2E). It was sutured to the external anal sphincter, ischiourethralis, periosteum of the ischial table/ischial border, sacrotuberous ligament, and rectal wall using 3–0 polydioxanone (PDS II, Ethicon, USA) (Figure 2F). The redundant distal portion of the flap was trimmed and removed. Both surgical fields were lavaged and closed routinely after bupivacaine infiltration. An active drain using a butterfly needle and a vacuum tube was placed at the perineal wound. Postoperative rectal palpation confirmed a well-reconstructed pelvic diaphragm supporting the rectal wall.

The patient recovered uneventfully from anesthesia, with a good appetite and no signs of lameness. A seven-day course of oral medication, including amoxicillin/clavulanic acid (12.5 mg/kg BW, BID; Amocla, Kuhnil, Korea), famotidine (0.5 mg/kg BW, BID; Famotidine, Nelson Korea, Korea), and meloxicam (0.1 mg/kg BW, once daily), was prescribed. A cold pack was recommended to be applied to the surgical sites (right perineum and right lateral thigh) twice daily for 3 days to alleviate potential acute pain, swelling, and inflammation.

**Figure 3:**
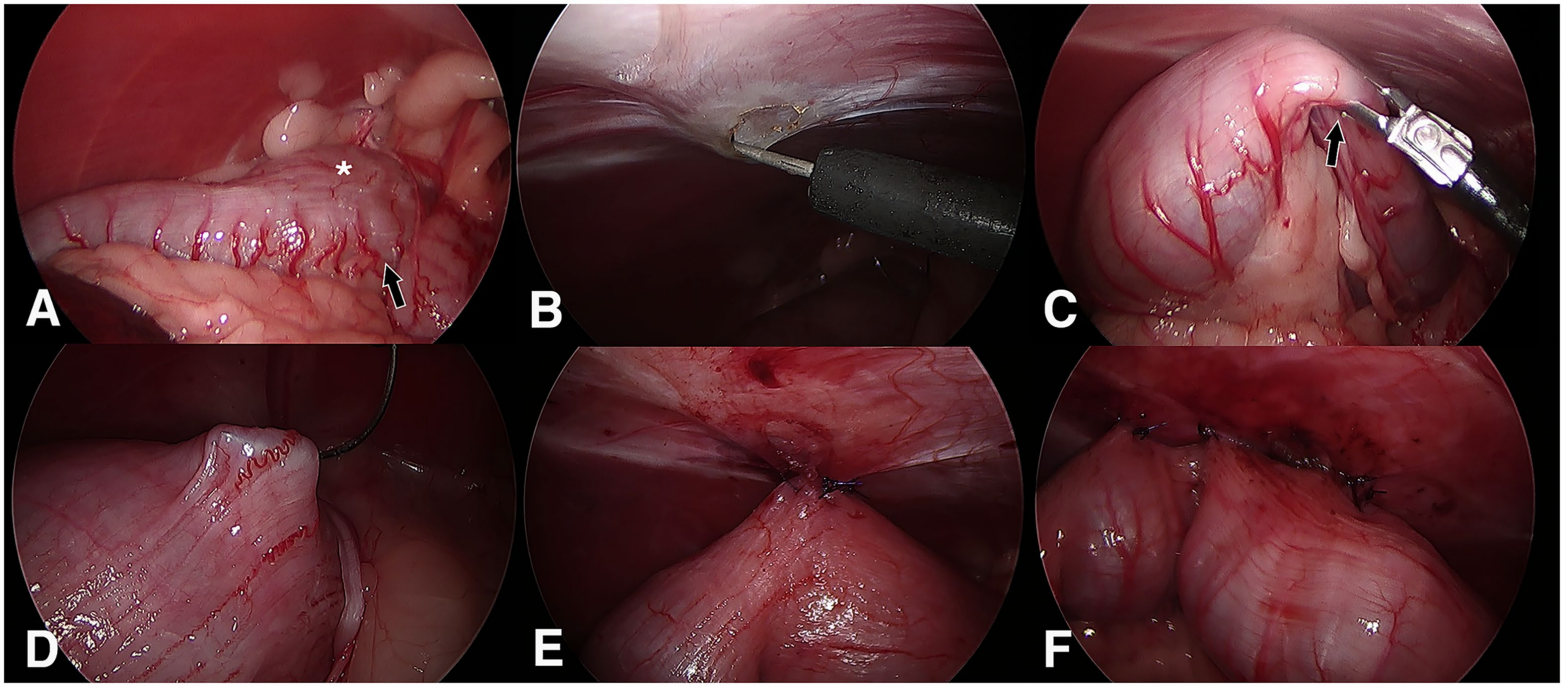
Laparoscopic colopexy for the treatment of rectal prolapse subsequent to perineal herniorrhaphy in a dog. **(A)** Exploration of the caudal abdomen and dilated distal colon (*), **(B)** peritoneal opening at the site to be pexied, **(C)** distal colon being retracted cranially and lifted up to the abdominal wall, **(D)** seromuscular bite of the colonic wall, **(E)** colopexy with two caudal sutures, **(F)** completed colopexy. The black arrows in panel **(A,C)** indicate the same location in the distal colon.

On the night of surgery, however, a 3 cm full-thickness RP developed. A temporary purse-string suture was performed after manual reduction, followed by laparoscopic colopexy the next day (postoperative day 1 [POD 1]). Three 5-mm ports were used: the primary port for the 5 mm, 0° telescope (1,488 HD, Stryker, Portage, Michigan, USA) was placed cranial to the umbilicus, and two instrumental ports were placed right paramedian, caudal to the primary port. Intra-abdominal pressure was maintained at < 10 mmHg throughout the procedure. The caudal abdomen and pelvic cavity were explored, and the distal colon was retracted cranially, revealing dilatation of the distal colon (Figure 3A). The selected site for colopexy was the intersection of the left paramedian line of the rectus abdominis muscle and the transverse line of the preputial end. A 2 cm longitudinal incision was made in the peritoneum using a J-hook for laparoscopic cauterization (Figure 3B). The colon was lifted to the abdominal wall, and 4 intracorporeal, simple interrupted sutures were placed under lower intra-abdominal pressure (6 mmHg) using 3–0 polydioxanone (PDS II, Ethicon, USA), taking care not to penetrate the colonic lumen (Figures 3C–F). The abdominal wall was sutured first, followed by the seromuscular layer of the colon. Sutures were placed in a single row from tail to head, with more than 5 mm between each suture. One suture (the third) failed due to tearing of the seromuscular layer of the colon during knot tightening and was reinforced with an additional percutaneous anchoring suture. The extracorporeal knot was buried subcutaneously through a deep stab incision in the skin. A mild bleeding point from puncturing a vessel on the serosal surface was easily managed by compression with a hemostatic agent (Surgicel®, Ethicon, USA).

**Figure 4:**
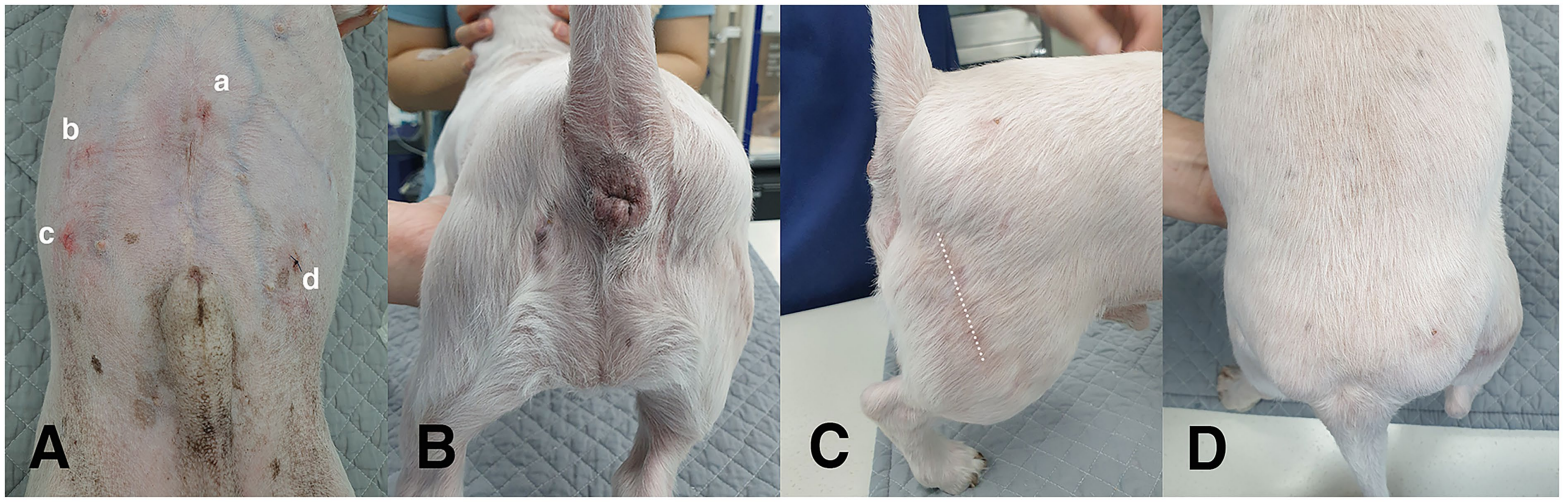
Portal sites for laparoscopic colopexy **(A)** and gross appearance at 3 weeks after fascia lata transposition for recurrent perineal hernia in a dog (**B**: caudal view, **C**: lateral view, **D**: dorsal view). a; camera port, b/c; instrumental ports, d; one simple interrupted skin suture to bury percutaneous anchoring suture, dotted line; scar of lateral skin incision to create fascia lata flap.

The patient recovered well, showing normal activity, appetite, urination, and defecation. The active drain at the perineal site and the fentanyl patch were removed on POD 3 and POD 4, respectively. The highest C-reactive protein (CRP) value was 82 mg/L (reference range: 0–20 mg/L) on POD 2, which subsided over time. The surgical sites appeared normal grossly (Figure 4). Abdominal radiography and ultrasonography performed on POD 10 and 17 confirmed that the colopexy site remained in its intended position, with no evidence of complications such as peritonitis, gastrointestinal dysfunction, or wound infection. Lactulose administration was discontinued on POD 20. The patient exhibited no signs of straining, and the feces were of normal length and shape. Rectal palpation revealed no abnormalities.

The patient remained hospitalized under institutional care after surgery until being adopted on POD 26. During a follow-up interview with the new owner, it was reported that the patient remained clinically well for 27 months after surgery, with no recurrence of PH or RP, and no associated clinical signs.

## 3 Discussion

Complications of perineal herniorrhaphy include wound dehiscence/infection (5–45%), seroma formation, persistent tenesmus (2–8%), urinary incontinence (4–8%), fecal incontinence (3–15%), neuropraxia, and recurrence (0–70%) (2, 5). Recurrence rates are influenced by various factors, including the surgeon’s experience, the chosen surgical method, a history of previous herniorrhaphy, local tissue strength, the type and tension of the suture material, predisposing factors, the severity of clinical signs, overactivity, surgical site infection, tenesmus, the development of fecal incontinence, underlying prostatic disease, and castration status (2, 16).

Although PH predominantly occurs in intact males, a definitive correlation between the disease and gonadal hormones, such as testosterone or estradiol, remains unestablished despite suspected hormonal influences (6). According to Hatch et al., a strong correlation exists between neutering status and the reccurrence of PH; specifically, dogs that underwent castration prior to PH development faced a 4.4-fold increase in recurrence odds, while those neutered concurrently with herniorrhaphy showed a protective effect (17). In contrast, Ferrari et al. identified no significant relationship between castration status and recurrence (16). The patient in the present case was a rescued, castrated male dog with no available medical history, presenting with bilateral perineal scars. A synthetic mesh inserted during a previous surgery was removed from the right side, while a subcutaneous foreign body, presumed to be the migrated mesh, was identified in the left perineal region. These findings suggest a chronic and recurrent condition following surgical repair involving bilateral synthetic mesh implantation, regardless of whether castration was performed prior to or concurrently with PH repair. The absence of a visible internal obturator muscle makes it unclear whether IOMT had been performed at the time of mesh implantation or whether the muscle had simply severely atrophied. Reported recurrence rates of PH after IOMT alone and IOMT combined with mesh implantation are up to 36% and approximately 12.5%, respectively (5, 18).

Regarding the repair of recurrent PH, an appositional suture technique could be used occasionally; however, transposition of peri-lesional or distant tissue, or implantation of synthetic or biomaterials, is usually required due to the massive atrophy or defect of the pelvic musculature. In this case of recurrent PH, conventional appositional herniorrhaphy and IOMT were not viable due to insufficient pelvic diaphragm muscles for proper apposition or suture anchoring. Thus, a salvage procedure was necessary, and FLT was selected over semitendinosus muscle transposition, as fascia closure provides a stronger repair compared to muscular closure, while utilizing fresh, robust tissue from a distant site rather than the compromised tissue at the lesion (4, 14). The ischiatic periosteum and sacrotuberous ligament were also used for anchoring the flap. The use of synthetic mesh was avoided due to the local inflammatory condition of the previous surgical site (though bacterial culture was omitted) and the surgeon’s preference for utilizing native tissue.

A fascia lata flap, rather than a graft, was created while preserving its muscular attachment to provide robust lateral fixation for the herniorrhaphy. If the flap length had been insufficient to cover the hernia defect extending to the medioventral aspect, its attachment could have been transected and used as a free graft. This technique has been reported to be easy to perform and handle, requiring minimal tissue dissection compared with semitendinosus muscle transposition, and resulting in low postoperative lameness scores (4, 14). Generally, fascia lata is large enough to cover hernia defects even in large-breed dogs, and in FLT, the base of the flap can provide lateral and ventral support successfully without suture placement (4, 14). Moreover, it does not form a nidus for persistent infection, and its autogenous nature minimizes the risks of immunologic foreign body reaction and associated surgical site infection, which are major causes of reconstruction failure in synthetic mesh implantation (4, 14). Autogenous tunica vaginalis grafting might be considered, but it was not possible in this case as this technique can be performed only in intact male dogs (19).

Despite this being the authors’ first experience with FLT, harvesting and handling the flap were accomplished with ease. However, the relatively narrow and caudally exposed surgical field positioned the skin incision near the cranial border of the biceps femoris muscle, slightly hindering the cranial dissection of the flap. A more cranially located and longer skin incision would have facilitated the flap creation. Therefore, it is recommended that the sterile surgical field be sufficiently wide, covering at least the entire surface of the lateral thigh. Alternatively, employing a hanging-leg technique during aseptic preparation and draping to expose the entire right hindlimb could also enhance the freedom of flap harvesting.

The characteristics of FLT, as mentioned above, make this technique suitable for recurrent PH (4), as observed in this case. Meanwhile, Sutthiprapa et al. suggested a sacroischial sling technique as another practical, low-complication alternative for recurrent PH that is cost-effective, straightforward, and comparable to IOMT while offering a shorter surgical time (7).

Large rectal sacculation and diverticulum—defined as rectal dilatation with or without disruption of the rectal muscularis layer leading to herniation of the mucosa through the defect—are common in chronic PH. These persistent lesions may induce excessive straining and tenesmus to expel impacted feces, ultimately resulting in PH repair failure and recurrence (2, 6–8, 20). In a recent study (16), histological evaluation of the rectal wall in patients with PH revealed significantly higher inflammation and fibrosis scores. Furthermore, PH recurrence was significantly associated with both a high rectal dilatation score—measured both immediately postoperatively and 60 days after herniorrhaphy—and a high fibrosis score. Accordingly, some surgeons recommend plication or lateral resection with inverting sutures prior to herniorrhaphy to prevent recurrence (6, 8, 9, 20). Intriguingly, in contrast to the aforementioned inability to defecate, another study also reported that patients developing postoperative fecal incontinence exhibited 3.4 times higher odds of PH recurrence (17).

Two previous studies using fascia lata for 21 complicated PHs in 18 dogs reported no recurrence of PH or development of RP during a mean follow-up of 5.8 months (*n* = 12 dogs, fascia lata graft for 15 PHs) and 24 months (*n* = 6 dogs, FLT for 6 PHs) (4, 14). Recurrent PH was identified in five among them, and rectal diseases included 14 rectal sacculations, 1 rectal deviation, and 3 rectal herniations. Although 2 dogs showed transient, manually reducible anal mucosal prolapses postoperatively, none of the 18 dogs required colopexy, cystopexy, or vasopexy. Accordingly, in the present patient, only herniorrhaphy using FLT was initially performed and colopexy was not carried out.

However, an acute RP developed on the night of FLT, even though the patient did not show marked signs of straining. In a previous study, colopexy, although ineffective in reducing rectal sacculation or diverticulum, restored the linear tubular structure of the colorectum and reduced rectal diameter, thereby decreasing fecal accumulation in the dilated rectum and consequently lowering the pressure on the pelvic diaphragm (5). Therefore, preemptive colopexy is recommended for complicated PH presenting with obvious rectal dilatation and recurrent RP (14). The development of RP in this case could be attributed to various factors that altered intra-pelvic pressure after herniorrhaphy without colopexy. These include the persistently dilated, deviated, and redundant rectum, despite the restoration of its intra-pelvic position, as well as postoperative discomfort or pain. Following the reconstruction of the pelvic diaphragm, the pressure previously dissipated through the hernia defect could no longer be released, subsequently leading to the RP. This necessitated surgical fixation to prevent further rectal expulsion driven by the increased intra-pelvic pressure. In terms of managing postoperative discomfort, implementing epidural anesthesia may serve as a valuable adjunctive strategy.

While a temporary purse-string suture alone might have been sufficient, a colopexy was chosen considering the complicated chronic condition of recurrent PH with rectal sacculation as previously described (20). Additionally, the patient was planned for adoption post-discharge, necessitating a more definitive treatment. When colopexy was performed alone, without excision or plication of the rectal sacculation, laparoscopy provided magnified visualization, which facilitated selective incorporation of only the seromuscular layer during colonic suturing in a minimally invasive manner. Lowering the intra-abdominal pressure during suturing was helpful in preventing overexpansion of the abdominal wall and excessive tension on the colon, particularly during placement of the first suture. Of the four simple interrupted sutures, one failed due to excessive tension caused by inadvertent manipulation; however, it was successfully secured with an additional percutaneous suture. To minimize such suture breakage, placing sutures in a cranio-caudal (head-to-tail) direction, rather than a caudo-cranial (tail-to-head) direction, could be beneficial. Conversion to laparotomy was not needed, and postoperative complications such as colonic perforation were not observed.

Laparoscopic or laparoscopic-assisted colopexy in dogs and cats has been reported in a limited number of publications (10, 12, 21, 22). Notably, significantly lower postoperative serum CRP levels have been observed in laparoscopic colopexy compared to conventional open colopexy in dogs, indicating less tissue inflammation (21). Conventionally, fixation sutures are recommended to be placed in two parallel rows with 5 to 8 sutures in each row, in an interrupted, continuous, or horizontal mattress suture pattern. Although laparoscopic colopexy requires skilled intracorporeal suturing, it has been reported to achieve stable adhesion at the pexy site on POD 30 in an experimental study using a total of six sutures arranged in two rows, with three sutures per row (21). However, in two previous reports using laparoscopic approaches, fixation with only 3 to 5 simple interrupted sutures in a single row was found to be sufficient (12, 22). Implementing five bites of a simple continuous suture pattern using barbed suture material was also effective in three cats, providing a stable adhesion (10).

As demonstrated in the present case, a combined surgical approach including herniorrhaphy and organopexy is highly effective for the management of complicated and recurrent PH. Although the two procedures were performed separately in this patient, when considering a combined surgical approach from the outset, Brissot et al. recommended a two-stage surgery with organopexy preceding herniorrhaphy to empty the perineal space, resolve rectal deviation, and facilitate herniorrhaphy (4, 5). This approach could benefit the herniorrhaphy procedure by providing advantages such as better anatomical visualization, easier suture placement, a decreased risk of trauma to the anal sphincter and caudal rectal nerves, and a subsequent decrease in postoperative fecal incontinence and recurrence rates. In their study of complicated PH in 41 dogs, IOMT and organopexy, including 41 colopexies, were performed, reporting a mean follow-up of 26.6 months, a success rate of 92%, and a recurrence rate of 10%; all PH relapses occurred within 6 months.

In this case report, the combination of FLT and laparoscopic colopexy for recurrent PH complicated by rectal sacculation and subsequent RP demonstrated a favorable outcome, with no recurrence of PH or RP observed up to 27 months postoperatively. FLT is an effective option for complicated PH characterized by a deficiency in the pelvic musculature for reconstruction, although the potential development of RP should be considered. Consequently, it is strongly recommended that colopexy be performed, either prior to or concurrently with FLT.

## Data Availability

The original contributions presented in the study are included in the article, further inquiries can be directed to the corresponding author.
